# Sex differences in the association between dietary choline intake and total bone mineral density among adolescents aged 12–19 in the United States

**DOI:** 10.3389/fnut.2024.1459117

**Published:** 2024-11-20

**Authors:** Hongyang Gong, Jiecheng Jiang, Seok Choi, Shaoqun Huang

**Affiliations:** ^1^Department of Oncology Surgery, Fuzhou Hospital of Traditional Chinese Medicine Affiliated to Fujian University of Traditional Chinese Medicine, Fuzhou, China; ^2^Department of Physiology, College of Medicine, Chosun University, Gwangju, Republic of Korea; ^3^College of Acupuncture and Orthopedics, Hubei University of Chinese Medicine, Wuhan, China

**Keywords:** dietary choline intake, bone mineral density, sex differences, NHANES, adolescents

## Abstract

**Background:**

While prior research has established a correlation between dietary choline intake and bone density in the elderly, the relationship in adolescents remains ambiguous. This study seeks to examine the association between dietary choline intake and bone density in American adolescents.

**Methods:**

Data from the National Health and Nutrition Examination Survey (NHANES) for 2005 to 2018 were used in this study, encompassing participants aged 12–19 years. The relationship between dietary choline intake and bone density was assessed using multivariate linear regression models and restricted cubic spline (RCS) models. Subgroup analyses were also performed to investigate differences across various subgroups.

**Results:**

3,800 participants with an average age of 15 years were included in this study. After adjusting for relevant confounding factors, a positive correlation was observed between dietary choline intake and total bone density in adolescents (95% CI: 0.03–0.17, *p* = 0.010). Gender-specific analysis indicated a significant positive correlation between dietary choline intake and total bone density in males (95% CI: 0.07–0.23, *p* < 0.001), while no significant correlation was found in females (95% CI: −0.19 to 0.09, *p* = 0.500). The stratified analysis revealed that the positive association was more pronounced in males and non-Hispanic whites (interaction *p* < 0.05). The restricted cubic spline model demonstrated a linear positive correlation between dietary choline intake and total bone density.

**Conclusion:**

This study demonstrates that dietary choline intake levels are positively correlated with bone density in adolescents, with this association being specific to males.

## 1 Introduction

Osteoporosis is a systemic bone disease caused by multiple factors, characterized by reduced bone density and quality, impaired bone microstructure, increased bone fragility, and a higher risk of fractures (1, 2). Epidemiological studies from China indicate that approximately 60.2 million people nationwide suffer from osteoporosis. Among those aged 50 and above, the prevalence is 6.46% in men and 29.13% in women, with these rates continuing to rise as the population ages (3, 4). The high fracture risk associated with osteoporosis significantly threatens individual quality of life and socioeconomic development (5), highlighting the critical importance of enhanced prevention and treatment efforts.

Adolescence is a crucial period for bone growth, with more than half of peak bone mass being formed during this time (6, 7). Research indicates that the peak bone mass achieved during adolescence is a key determinant of fracture and osteoporosis risk in later life (8, 9). Interventions during this period can effectively prevent osteoporosis and fractures in adulthood. While genetics play a vital role in determining peak bone mass, dietary nutrition can modify the genetic potential for bone growth and is essential for the skeletal development of adolescents (10–12).

Mojgan and colleagues have shown that nutrients such as calcium, vitamin D and dietary antioxidants play an important role in preventing chronic diseases such as blood pressure and bone disorders (13, 14). Choline, an essential nutrient, participates in numerous physiological functions in the human body, such as acetylcholine synthesis, neurotransmission, cell membrane stability, and lipid metabolism, all crucial for maintaining health and well-being (15, 16). Existing research has established correlations between dietary choline levels and the risk of osteoporosis in older adults (17). However, studies examining the relationship between dietary choline and bone density are still limited, particularly in adolescent populations.

Therefore, this study utilized the National Health and Nutrition Examination Survey (NHANES) database to investigate the relationship between dietary choline intake and bone density in American adolescents.

## 2 Methods

The National Health and Nutrition Examination Survey (NHANES) is a nationwide health survey conducted jointly by the Centers for Disease Control and Prevention (CDC) and the National Center for Health Statistics (NCHS). It employs a complex sampling design to select participants and collects data through questionnaires, physical examinations, and laboratory tests. NHANES aims to evaluate the health and nutritional status of the non-institutionalized U.S. population. Both the research data and survey methods of NHANES are openly accessible on its website. The research protocol has received ethical approval from a review board, and all participants have provided written informed consent.

### 2.1 Study design and population

**Figure 1:**
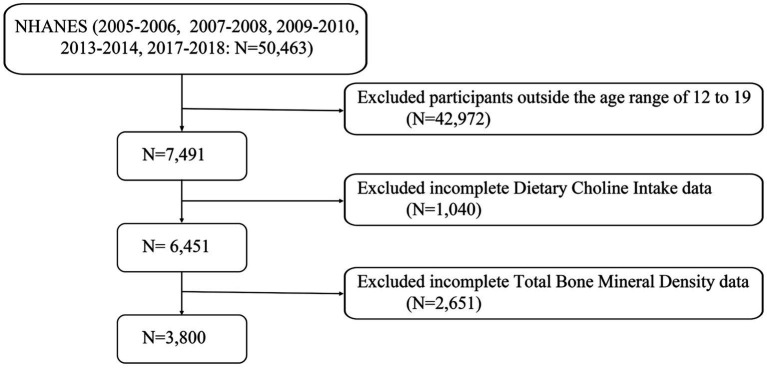
A flow diagram of eligible participant selection in the National Health and Nutrition Examination Survey.

This study utilized data collected from the National Health and Nutrition Examination Survey (NHANES) spanning from 2005 to 2018, excluding periods with missing bone density data in 2011–2012 and 2015–2016. Initially, a total of 50,463 participants were considered. Following the exclusion of individuals outside the 12–19 age range (*N* = 42,972), those with missing dietary choline data (*N* = 1,040), and those lacking total bone density data (*N* = 2,651), the final analysis included 3,800 participants (Figure 1).

### 2.2 Assessment of dietary choline intake

Dietary choline intake was assessed using data from two 24-h dietary recall interviews. The first interview occurred at a mobile examination center, followed by a second interview conducted via phone several days later. Nutrient and micronutrient intake was calculated using the United States Department of Agriculture (USDA) database, with the analysis based on the average intake from the two recall days.

### 2.3 Evaluation of total bone mineral density

Dual-energy X-ray absorptiometry (DXA) is regarded as the primary method for measuring bone density in humans due to its advantages of speed, simplicity, and minimal radiation exposure. Total bone density was measured using a Hologic QDR 4500A fan-beam densitometer by trained radiologic technologists (18). Detailed measurement procedures are available on the NHANES website.

### 2.4 Covariates

NHANES has gathered extensive data on demographic and dietary factors, encompassing age, sex, race, family income-to-poverty ratio, obesity, physical activity level, daily energy intake, dietary carbohydrates, fiber, fat, calcium, and phosphorus intake. Definitions of covariates can be found in Supplementary Table S1.

### 2.5 Statistical analysis

Given the complex, multi-stage, probability sampling design of the NHANES database, sample weights were applied to combine data across multiple survey cycles to ensure that results better represent the characteristics of the entire U.S. population. In this study, we utilized the WTDRD2 weight (weight for participants who completed the second 24-h dietary recall interview).

Initially, descriptive statistics were used to analyze population characteristics, with continuous variables presented as means ± standard deviations and categorical variables as frequencies and percentages. Weighted *t*-tests were employed for comparing continuous variables, while weighted chi-square tests were used for categorical variables (19). Multiple linear regression models were used to analyze the correlation between dietary choline intake and adolescent bone density. Further analyses were conducted separately for males and females to validate these relationships. Three regression models were employed to control for confounding factors: Model 1 was unadjusted, Model 2 adjusted for age, sex, race, and family income-to-poverty ratio, and Model 3 additionally adjusted for obesity, physical activity, daily energy intake, dietary carbohydrates, fiber, fat, calcium, and phosphorus intake.

Restricted cubic spline (RCS) models were employed to analyze whether there was a non-linear relationship between choline intake and adolescent bone density. Subgroup analyses based on key covariates (e.g., gender, race, household income, obesity, and physical activity) were conducted to examine possible interactions between dietary choline intake and different stratification factors. All statistical analyses were performed using R version 4.2.2 software, with statistical significance set at *p* < 0.05.

## 3 Results

### 3.1 Characteristics of the participants

Table 1 outlines the baseline characteristics of participants, encompassing a total of 3,800 subjects included in our study. The average age of the study cohort was 15.47 ± 2.22 years, with a predominant representation of non-Hispanic White individuals (1,132 cases, 62%). Compared to females, males exhibited a higher proportion engaged in vigorous physical activity (*p* < 0.05). Moreover, male participants demonstrated higher daily energy intake, dietary choline, carbohydrates, fiber, fat, calcium, and phosphorus intake compared to females (*p* < 0.001). Regarding bone density, males also exhibited significantly higher total bone density than females (*p* < 0.001).

**Table 1:** Baseline characteristics of all participants were stratified by gender.

Characteristic	Overall, *N* = 3,800 (100%)	Male, *N* = 2,004 (52%)	Female, *N* = 1,796 (48%)	*p* value
Age (mean (SD))	15.47 (2.22)	15.49 (2.26)	15.43 (2.18)	0.624
Race (%)				0.593
Non-Hispanic White	1,132 (62%)	610 (63%)	522 (62%)	
Mexican American	1,080 (12%)	569 (12%)	511 (11%)	
Non-Hispanic Black	1,074 (14%)	558 (14%)	516 (15%)	
Other	514 (12%)	267 (12%)	247 (12%)	
PIR (%)				0.165
Not poor	2,138 (56.3%)	1,149 (57.3%)	989 (55.1%)	
Poor	1,421 (37.4%)	722 (36.1%)	699 (38.9%)	
Unknown	241 (6.3%)	133 (6.6%)	108 (6%)	
Obesity (%)				0.779
No	3,224 (84.8%)	1,723 (85.9%)	1,501 (86.9%)	
Yes	569 (15%)	276 (13.9%)	293 (13%)	
Unknown	7 (0.2%)	5 (0.2%)	2 (0.1%)	
Physical activity (%)				**0.034**
Inactive	1,238 (32.6%)	665 (33.2%)	573 (31.9%)	
Active	2,017 (53.1%)	1,146 (57.2%)	871 (48.5%)	
Unknown	545 (14.3%)	193 (9.6%)	352 (19.6%)	
Energy, kcal/d (mean (SD))	2,167.46 (931.03)	2,484.49 (1,013.45)	1,821.33 (680.18)	**<0.001**
Dietary Carbohydrate, g/d (mean (SD))	281.25 (124.80)	320.41 (136.00)	238.49 (94.28)	**<0.001**
Dietary fiber, g/d (mean (SD))	13.95 (7.13)	15.46 (7.78)	12.30 (5.91)	**<0.001**
Total fat, g/d (mean (SD))	81.31 (40.94)	92.68 (45.06)	68.90 (31.54)	**<0.001**
Total calcium, mg/dL (mean (SD))	9.66 (0.32)	9.74 (0.32)	9.57 (0.29)	**<0.001**
Phosphorus, mg/dL (mean (SD))	4.42 (0.69)	4.54 (0.74)	4.28 (0.60)	**<0.001**
Dietary choline intake, mg/d (mean (SD))	280.68 (146.07)	326.58 (162.31)	230.56 (105.19)	**<0.001**
Total BMD, g/cm^2^ (mean (SD))	0.98 (0.16)	1.01 (0.17)	0.95 (0.13)	**<0.001**

Mean (SD) for continuous variables: the *p* value was calculated by the weighted linear regression model.

Percentages for categorical variables: the *p* value was calculated by the weighted chi-square test.

PIR, Ratio of family income to poverty; BMD, Bone Mineral Density.

### 3.2 Association between dietary choline intake and total BMD

We employed three linear regression models to investigate the independent relationship between dietary choline intake and adolescent bone density (Table 2). In Model 1, choline intake exhibited a significant positive association with adolescent bone density (*β*: 0.18, 95% CI: 0.14–0.22), which remained significant in the minimally adjusted Model 2 (β: 0.08, 95% CI: 0.04–0.11) and fully adjusted Model 3 (β: 0.10, 95% CI: 0.03–0.17). Stratifying choline intake into quartiles yielded consistent findings.

**Table 2:** Association between dietary choline intake and total BMD in all participants.

Choline intake	Model 1 [Beta (95% CI)]	*p*-value	Model 2 [Beta (95% CI)]	*p*-value	Model 3 [Beta (95% CI)]	*p*-value
Continuous (per 1 g)	0.18 (0.14, 0.22)	<0.001	0.08 (0.04, 0.11)	<0.001	0.10 (0.03, 0.17)	0.010
Quartile						
Q1	1 (ref.)		1 (ref.)		1 (ref.)	
Q2	0.01 (−0.02, 0.02)	>0.9	0.01 (−0.02, 0.02)	0.800	0.01 (−0.02, 0.04)	0.500
Q3	0.02 (0.01, 0.04)	0.047	0.01 (−0.02, 0.02)	0.700	0.01 (−0.01, 0.03)	0.500
Q4	0.06 (0.05, 0.08)	<0.001	0.03 (0.01, 0.04)	<0.001	0.03 (0.01, 0.06)	0.007
*p* for trend	<0.001		<0.001		0.007	

Model 1: no covariates were adjusted.

Model 2: age, gender, PIR, and race were adjusted.

Model 3: age, gender, PIR, race, obesity, physical activity, energy, dietary carbohydrates, dietary fiber, total fat, total calcium, and phosphorus were adjusted.

PIR, Ratio of family income to poverty; CI, confidence interval; BMD, Bone Mineral Density.

**Figure 2:**
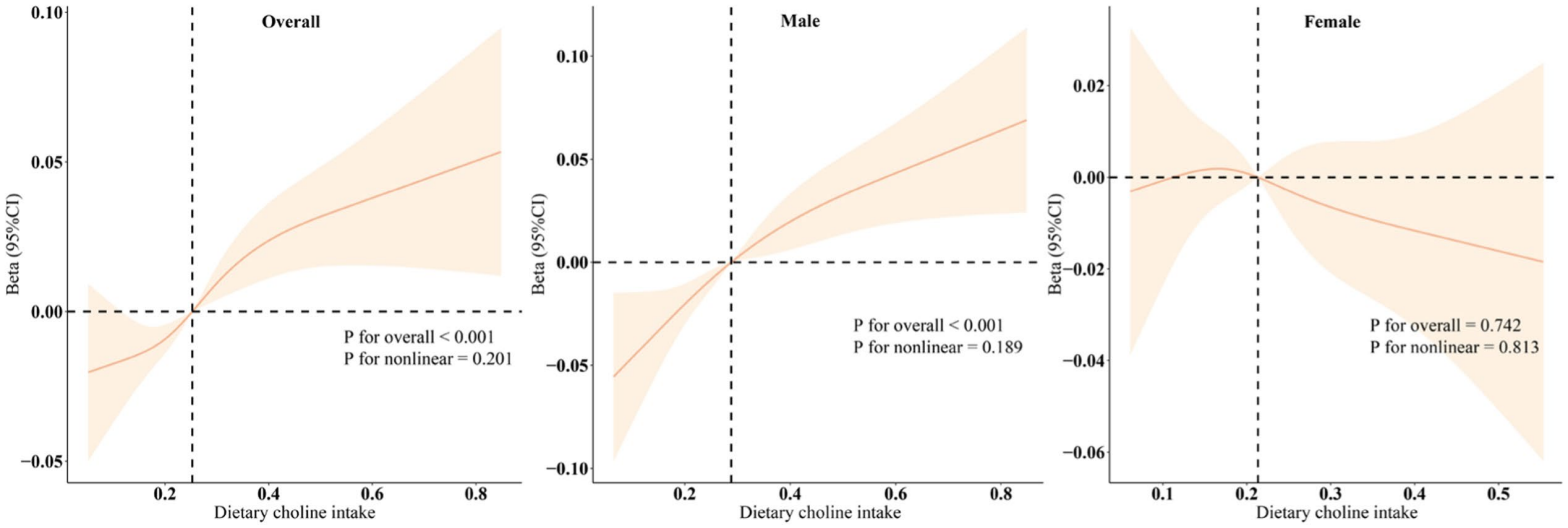
Dose–response relationships between dietary choline intake and total BMD. Beta (solid lines) and 95% confidence levels (shaded areas) were adjusted for age, gender, PIR, race, obesity, physical activity, energy, dietary carbohydrates, dietary fiber, total fat, total calcium, and phosphorus.

Upon performing separate regression analyses for males and females (Table 3), we observed that the positive correlation between choline intake and bone density was exclusively significant among male participants (*p* < 0.001), whereas no significant correlation was found among females (*p* > 0.05). Quartile analysis further supported these results. Restricted cubic spline (RCS) models demonstrated a linear positive association between dietary choline intake and adolescent bone density, which was statistically significant solely in male participants (*p* < 0.001) (Figure 2).

**Table 3:** Sex-specific association between dietary choline intake and total BMD.

Choline intake	Model 1 [Beta (95% CI)]	*p*-value	Model 2 [Beta (95% CI)]	*p*-value	Model 3 [Beta (95% CI)]	*p*-value
Male						
Continuous (per 1 g)	0.18 (0.13, 0.24)	<0.001	0.08 (0.03, 0.12)	<0.001	0.15 (0.07, 0.23)	<0.001
Quartile						
Q1	1 (ref.)		1 (ref.)		1 (ref.)	
Q2	0.02 (−0.02, 0.06)	0.400	0.01 (−0.01, 0.04)	0.300	0.02 (−0.01, 0.04)	0.200
Q3	0.05 (0.01, 0.08)	0.009	0.02 (0.01, 0.05)	0.045	0.03 (0.01, 0.06)	0.011
Q4	0.08 (0.05, 0.11)	<0.001	0.04 (0.02, 0.06)	<0.001	0.06 (0.03, 0.08)	<0.001
*p* for trend	<0.001		<0.001		<0.001	
Female						
Continuous (per 1 g)	−0.03 (−0.10, 0.03)	0.400	−0.02 (−0.08, 0.05)	0.600	−0.05 (−0.19, 0.09)	0.500
Quartile						
Q1	1 (ref.)		1 (ref.)		1 (ref.)	
Q2	−0.01 (−0.04, 0.01)	0.200	0.01 (−0.03, 0.02)	0.800	0.01 (−0.03, 0.04)	0.700
Q3	−0.03 (−0.05, −0.01)	0.009	−0.02 (−0.04, 0.08)	0.076	−0.03 (−0.06, 0.01)	0.080
Q4	0.01 (−0.02, 0.03)	0.800	0.01 (−0.02, 0.03)	0.600	0.01 (−0.04, 0.06)	0.800
*p* for trend	0.800		0.600		0.800	

Model 1: no covariates were adjusted.

Model 2: age, PIR, and race were adjusted.

Model 3: age, PIR, race, obesity, physical activity, energy, dietary carbohydrates, dietary fiber, total fat, total calcium, and phosphorus were adjusted.

PIR, Ratio of family income to poverty; CI, confidence interval; BMD, Bone Mineral Density.

### 3.3 Subgroup analysis

**Figure 3:**
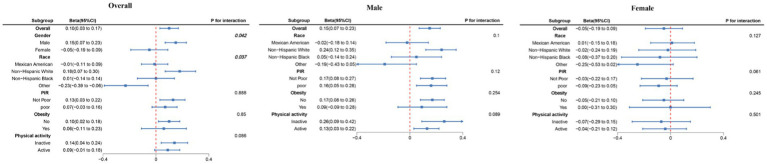
Subgroup analysis between dietary choline intake and total BMD. Beta was calculated as per 1 g increase in PHDI. Analyses were adjusted for age, gender, PIR, race, obesity, physical activity, energy, dietary carbohydrates, dietary fiber, total fat, total calcium, and phosphorus.

Subgroup analysis revealed a positive correlation between dietary choline intake and adolescent bone density across most subgroups (Figure 3). This correlation was notably stronger among males and non-Hispanic White individuals, aligning consistently with the results of our regression analysis.

## 4 Discussion

This study explored the relationship between dietary choline intake and bone density among American adolescents. We observed a significant positive correlation between dietary choline intake and adolescent bone density. In fully adjusted linear models, higher choline intake was consistently associated with higher bone density, primarily observed in male participants. Subsequent subgroup analyses further highlighted that this association was notably stronger among males and non-Hispanic White individuals.

As the population ages, the prevalence of osteoporosis continues to rise, accompanied by a heightened risk of fractures, imposing significant burdens on both households and socio-economic development (20, 21). Peak bone mass (PBM) is recognized as a determinant of osteoporosis and brittle bone fractures later in life (22), with the majority of bone mass accumulating during adolescence. Intervening effectively during this period to promote bone growth may hold crucial implications for preventing osteoporosis in old age (23–25). Dietary intake plays a critical role in bone growth among adolescents, as adequate consumption of key nutrients facilitates healthy skeletal development and maximizes bone density, thereby reducing the risks of osteoporosis and fractures.

Choline is an essential nutrient in the human body, serving as a crucial component of phosphatidylcholine and a precursor to the important neurotransmitter acetylcholine. It plays a significant role in maintaining cellular membrane structure and function, neurotransmission, lipid metabolism, regulation of intestinal microbiota, fetal development, and methylation processes (15, 26, 27). Recent studies have explored the relationship between choline and bone density. A health study involving 4,632 participants in the Hordaland region found a positive correlation between dietary choline intake and bone density, particularly strong among elderly men (17). Similarly, analysis of NHANES data from 2005 to 2010 revealed that increased daily dietary choline intake effectively reduces osteoporosis risk in elderly individuals (28). However, research on the relationship between dietary choline and bone density remains limited, primarily focusing on elderly populations. Currently, there is a lack of clinical reports on the impact of dietary choline on skeletal health in adolescents.

Our research has found a positive correlation between dietary choline intake and bone density in adolescents, although the specific mechanisms remain unclear. Based on existing research, we hypothesize several potential mechanisms. Firstly, dietary choline serves as a crucial precursor for acetylcholine synthesis. Acetylcholine, as the primary neurotransmitter of the parasympathetic nervous system (PSNS), activates the neuro-skeletal axis. This regulation through the cholinergic system promotes osteoblast proliferation and osteoclast apoptosis, thereby maintaining bone homeostasis (29–31). Additionally, acetylcholine has been shown to stimulate growth hormone release by activating α2-adrenergic receptors in the hypothalamus or nicotinic cholinergic receptors, further promoting skeletal health (32). Secondly, choline can be oxidized to betaine, which serves as a methyl donor for DNA epigenetic regulation (33). Research by Jing et al. (34) suggests that betaine induces osteogenic differentiation of bone marrow mesenchymal stem cells *in vitro* and inhibits adipogenic differentiation. Moreover, betaine has been found to enhance bone strength by reducing plasma homocysteine levels (35). Furthermore, choline’s regulation of lipid metabolism may also promote bone growth. Phosphatidylcholine, produced from choline, is essential for chylomicron formation in the intestine and serves as a preferred substrate for probiotic lactobacilli (36, 37). Through modulation of gut microbiota, it affects lipid metabolism, exerting dual regulatory effects on bone growth: increasing bone mass by adjusting lipid levels and mitigating bone loss caused by inflammation and oxidative damage due to lipid imbalance (38–40). However, these mechanisms require further investigation for clarification.

It is noteworthy that our regression analyses conducted separately for male and female participants revealed a positive correlation between dietary choline intake and bone density exclusively among adolescent males, a finding consistent across subgroup analyses. We speculate this outcome may be linked to estrogen secretion. Endogenous choline synthesis in the body primarily relies on phosphatidylethanolamine N-methyltransferase (PEMT) activity in the liver, which estrogen can enhance (41, 42). Increased estrogen secretion during puberty in females stimulates PEMT production, thereby elevating endogenous choline biosynthesis rates. This may explain why adolescent females are less susceptible to the effects of choline deficiency.

This study utilized nationally representative large-scale follow-up data to analyze the relationship between dietary choline intake and bone density in a cohort of American adolescents. We adjusted for demographic, clinical, and laboratory covariates to explore the impact of gender differences on the association between dietary choline and bone density. Overall, our findings underscore the significant role of dietary choline intake in enhancing bone density specifically among adolescent males. These results provide valuable insights for managing adolescent skeletal health. However, several limitations should be noted: (1) The observational study design precludes establishing causal relationships; (2) Dietary choline intake is estimated rather than directly measured and may not accurately reflect actual intake, and individual dietary habits may change during long-term follow-up; in addition, multivitamins from other supplements are commonly used by young people, and all of the above may have some influence on the results of this study. In future studies, we will consider controlling more fully for the effects of supplement use on study outcomes by collecting additional information on supplement use or by using a prospective design. (3) Despite extensive adjustment for relevant confounders, residual confounding factors may still exist. Therefore, in future studies, we will consider using more stringent methods, such as restriction strategies, to further mitigate the impact of these primary confounding factors and improve our findings’ interpretability and scientific validity. (4) For the age range of participants in this study, BMI classification should be based on BMI Z-scores, which can be standardized and adjusted for age and gender to more accurately reflect differences in growth and development. Therefore, this study may have some bias in classifying the obesity status of some participants. We will use BMI Z-scores to improve the accuracy of BMI classification in future studies in adolescent populations to further explore the effects of nutritional intake on bone health.

## 5 Conclusion

In conclusion, our study identified a positive correlation between dietary choline intake and bone density in adolescents, which was observed exclusively in males. This suggests that supplementing dietary choline may promote skeletal health development in male adolescents. Further epidemiological and laboratory research is needed to validate our findings.

## Data Availability

The datasets presented in this study can be found in online repositories. The names of the repository/repositories and accession number(s) can be found in the article/Supplementary material.
