# Supplementary material for: Sex differences in the association between dietary choline intake and total bone mineral density among adolescents aged 12–19 in the United States
Source: Front Nutr. 2024 Nov 20;11:1459117. doi: 10.3389/fnut.2024.1459117 (PMC11614608; doi:10.3389/fnut.2024.1459117)
Supplement: Supplementary file 1 [file Table_1.DOCX]

**Supplementary Material**

**Table S1.** Description of covariates

| Covariates | Description in NHANES |
| --- | --- |
| Age | 12 to 19 years old |
| Gender | Male and Female |
| Race | Mexican American, Non-Hispanic Black, Non-Hispanic White, Other Race |
| PIR | Poor: <1.3; Not Poor:>=1.3 |
| Obesity | Yes: BMI>=30 |
| Physical activity | Active physical activity was defined as >599 MET, or >149 min of moderate physical activity, or >74 min of vigorous physical activity |
| Energy, dietary carbohydrates, dietary fiber, and total fat | Obtained from two 24-hour dietary recall interviews and averaged over two days. |
| Total calcium and phosphorus | The methodology for the laboratory tests is described in detail on the NHANES website (http://www.cdc.gov/nchs/nhanes/index.htm). |

PIR, Ratio of family income to poverty
